# Synthesis, Crystal Structure, and Computational Methods of Vanadium and Copper Compounds as Potential Drugs for Cancer Treatment

**DOI:** 10.3390/molecules25204679

**Published:** 2020-10-14

**Authors:** Nidia D. Corona-Motolinia, Beatriz Martínez-Valencia, Lisset Noriega, Brenda L. Sánchez-Gaytán, Miguel Ángel Méndez-Rojas, Francisco J. Melendez, María Eugenia Castro, Enrique González-Vergara

**Affiliations:** 1 Centro de Química del Instituto de Ciencias, Benemérita Universidad Autónoma de Puebla, 18 sur y Av. San Claudio, Col. San Manuel, Puebla C. P. 72570, Mexico; nidia.corona@alumno.buap.mx (N.D.C.-M.); beatriz.mvalencia@alumno.buap.mx (B.M.-V.); brenda.sanchez@viep.com.mx (B.L.S.-G.); 2 Facultad de Ciencias Químicas, Benemérita Universidad Autónoma de Puebla, 18 sur y Av. San Claudio, Col. San Manuel, Puebla C. P. 72570, Mexico; lisset.noriegad@alumno.buap.mx (L.N.); francisco.melendez@correo.buap.mx (F.J.M.); 3 Departamento de Ciencias Químico Biológicas, Universidad de las Américas. Puebla, Sta. Catarina Mártir, Cholula Puebla C.P. 72820, Mexico; miguela.mendez@udlap.mx

**Keywords:** copper, vanadium, cancer treatment, Hirshfeld surfaces, AIM analysis, molecular docking

## Abstract

Transition metal-based compounds have shown promising uses as therapeutic agents. Among their unique characteristics, these compounds are suitable for interaction with specific biological targets, making them important potential drugs to treat various diseases. Copper compounds, of which Casiopeinas^®^ are an excellent example, have shown promising results as alternatives to current cancer therapies, in part because of their intercalative properties with DNA. Vanadium compounds have been extensively studied for their pharmacological properties and application, mostly in diabetes, although recently, there is a growing interest in testing their activity as anti-cancer agents. In the present work, two compounds, [Cu(Metf)(bipy)Cl]Cl·2H_2_O and [Cu(Impy)(Gly)(H_2_O)]VO_3_, were obtained and characterized by visible and FTIR spectroscopies, single-crystal X-ray diffraction, and theoretical methods. The structural and electronic properties of the compounds were calculated through the density functional theory (DFT) using the Austin–Frisch–Petersson functional with dispersion APFD, and the 6-311 + G(2d,p) basis set. Non-covalent interactions were analyzed using Hirshfeld surface analysis (HSA) and atom in molecules analysis (AIM). Additionally, docking analysis to test DNA/RNA interactions with the Casiopeina-like complexes were carried out. The compounds provide metals that can interact with critical biological targets. In addition, they show interesting non-covalent interactions that are responsible for their supramolecular arrangements.

## 1. Introduction

**Figure 1:**
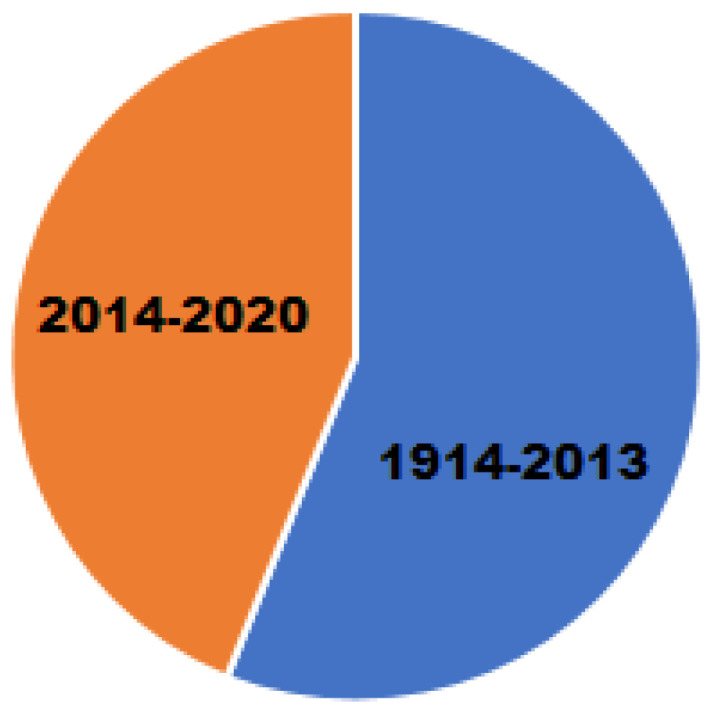
Papers that deal with copper related to Cancer, as found in the PubMed database.

Cancer represents a significant public health problem worldwide. Considering there were about 18.1 million new cancer cases and 9.6 million cancer deaths worldwide in 2018, it is relevant to find low-cost and safe alternatives to combat it [1]. As the second leading cause of death in the Americas, cancer caused 1.3 million deaths in 2018, and 3.7 million new cases were reported. By 2030, the number of cancer cases is estimated to increase by 32%, exceeding 5 million new cases due to the aging of the population and the epidemiological transition in Latin America and the Caribbean (PAHO/WHO) [2]. Metal-containing therapeutic agents comprise a fundamental class of drugs for treating tumors. Although many metal-containing drugs based on gold, ruthenium, gallium, titanium, iron, and copper are in preclinical and clinical trials phases I and II [3,4,5,6,7], cisplatin and also second- and third-generation platinum coordination compounds (carboplatin, oxalyplatin, and picoplatin) are still the most effective antitumor agents used in clinical practice [7]. However, the clinical use of platinum-based drugs entails many severe side effects, such as nephrotoxicity [8], neurotoxicity [9], and also ototoxicity and myelosuppression [10]. It is assumed that antitumor drugs based on endogenous metals (Co, Cu, Zn, and Fe) are less toxic than platinum analogs [11]. Many useful applications of these compounds require that the complex binds to DNA specifically through an intercalative mode with the ligand intercalating into adjacent base pairs of DNA molecules. Indeed, the square pyramidal structure characteristic of this type of copper(II) complexes provides an optimal geometry for their interaction with DNA strands, rendering them as alternatives to platinum-based anti-cancer drugs with the advantage that copper is better tolerated and can be more easily handled than other transition metals. Much attention has been paid to complexes containing symmetric aromatic ligands, such as 1,10-phenanthroline and 2,2′-bipyridine, and how they interact with DNA [12,13,14,15,16,17,18,19,20]. Important advances have been made in the field since Sigman et al. (1979) showed that [Cu(phen)_2_]^+^ complexes can inhibit DNA or RNA polymerase activities and can induce the scission of DNA strands in the presence of H_2_O_2_ or thiols [21]. Kwik et al. (1980) first synthesized and characterized a series of ternary complexes, including [Cu(phen)L]·nH_2_O, [Cu(bipy)L]·nH_2_O, [Cu(phen)LX]·nH_2_O and [Cu(bipy)LX]·nH_2_O [22]. From then on, several studies have shown that the geometry exhibited by the metal center, coupled with planar bidentate ligands, provides an optimal spatial arrangement to interact with many biological molecules providing the compounds with antitumoral and antiviral properties. Additionally, copper(II) complexes containing 1,10-phenanthroline can also function as chemical nucleases [23]. This important mechanism occurs by a Cu (II)/Cu (I) redox reaction that catalyzes the formation of reactive oxygen species (ROS). The structure of this type of complex comprises a five-coordinate copper(II) center displaying a distorted square pyramidal geometry, which exhibits an efficient DNA cleavage activity at micromolar concentrations in the presence of ascorbate with hydroxyl radicals as the active species [24]. To point out the recent relevance of the subject, the graph in Figure 1 shows in blue 100 years of previous reports, and in orange, the reports in the last six years in PubMed, using the keywords Copper and Cancer.

Vanadium, on the other hand, has well-documented therapeutic properties. At the same time, most of the vanadium pharmacological properties have been analyzed in treating diabetes mellitus. However, several studies have also found that vanadium can be used for cancer treatment since it induces cell apoptosis, displaying cytotoxic and antiproliferative effects [25]. In fact, there are several similarities in some metabolic pathways used by diabetes mellitus and cancer [26,27,28,29,30,31,32]. Vanadate and oligovanadates specifically act as anti-cancer drugs, as shown recently for studies with pancreatic cancer and malignant melanoma [33,34,35]. Additionally, the compound bis(4,7-dimethyl-1,10-phenanthroline) sulfatooxidovanadium(IV), known as Metvan, has been recently used in cytotoxicity studies with human osteosarcoma (MG-63) and human colorectal adenocarcinoma (HT-29) cell lines, displaying impaired cell viability of both cancer cell lines in a low concentration range (0.25–5.0 μM) [36]. Our group recently reported three new cyclotetravanadates as the first set of V/Cu heterobimetallic compounds with the potential to be used as metallodrugs in cancer treatment [37,38]. Here, we report the synthesis and experimental–theoretical characterization of two new compounds resulting from our search to complete the cyclotetravanadate copper complexes family. Interestingly, although the same procedure was followed in both syntheses, subtle differences in the mixture pot resulted in different compounds. Two new copper complexes, one without vanadium and the other with vanadium as a metavanadate ion, were obtained. The compounds were studied using computational methods. In docking experiments, the results show moderate interactions with DNA fragments and RNA. However, solid-state structure studies showed a variety of interesting non-covalent interactions responsible for their supramolecular crystal structure.

## 2. Results 

**Figure 2:**
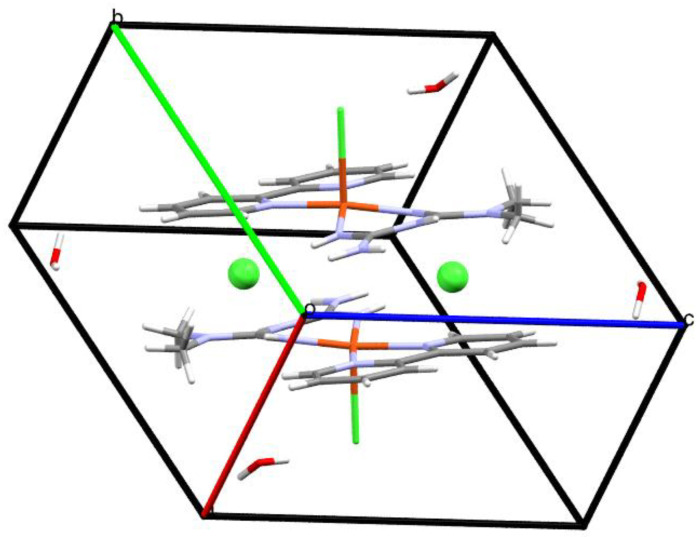
Unit cell content of Compound **1** using capped stick representation.

Crystal data, data collection, and structure refinement details are summarized in Table 1. Fractional atomic coordinates and isotropic or equivalent isotropic displacement parameters (Å) and geometric parameters (Å) for [Cu(Metf)(bipy)Cl]Cl·2H_2_O (Compound **1**) and [Cu(Impy)(Gly)(H_2_O)]VO_3_, (Compound **2**), are presented in Tables S1–S3 and S4–S6 respectively, in the Supplementary Material section. Compound **1** contains in its structure a central copper(II) atom that shows a square pyramidal geometry (τ_5_ = 0.073), to which the NH_2_ groups of metformin and the two N donors of bipyridine are coordinated in basal positions. A chlorine atom occupies the compound’s apical position, and the other chlorine atom is not coordinated but acts as a counter ion neutralizing the charge of the copper complex. Two formula units are contained in the unit cell, as shown in Figure 2.

**Figure 3:**
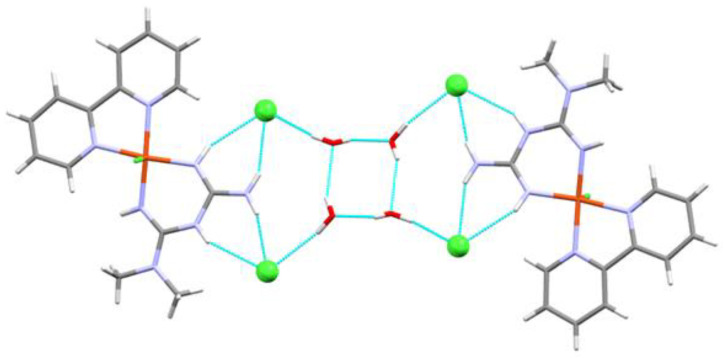
Capped stick representation of the supramolecular dimer of Compound **1**, showing hydrogen bonds and chloro contacts. Chlorine atoms are shown in ball and stick representation.

In Figure 3, a cyclic water (H_2_O)_4_ motif is surrounded by the copper complexes generating supramolecular dimers held together by halogen bridges, which also connect with neighbors through halogen contacts. The hydrogen bonds involved in making the cyclic water motif contain four donor atoms and four acceptors in an almost planar arrangement.

The distances and angles are summarized in Table 2. Not only are there many hydrogen bonds and halogen contacts, but also the rings of neighbor complexes are at a distance of 3.777 Å, close enough for π–π interactions, as seen in Figure S1. 

**Figure 4:**
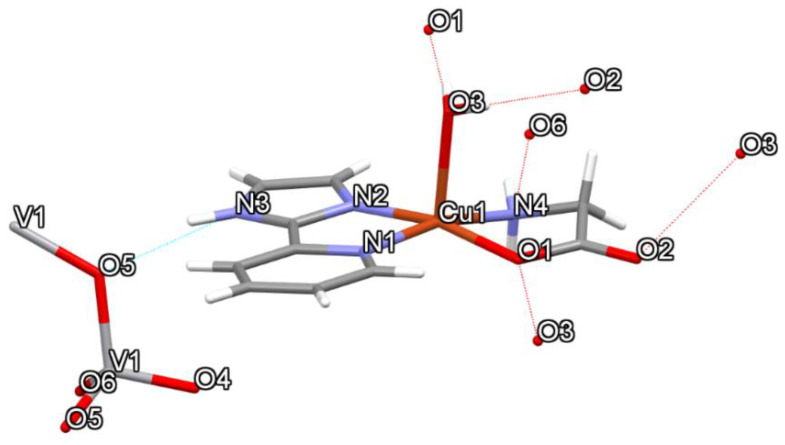
The capped stick representation of Compound **2** shows the most important hydrogen bonds involved in the interactions with the nearest neighbors.

**Figure 5:**
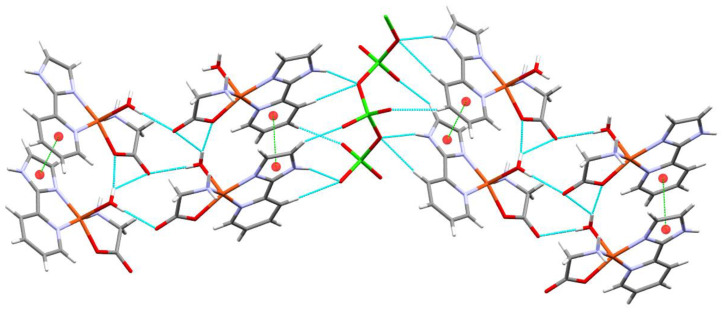
Capped stick representation of eight cationic units of Compound **2**, showing the hydrogen bonds and the π–π interactions between them.

Compound **2** contains a central copper(II) atom that also has a square pyramidal geometry (τ_5_ = 0.004), where the amino N and carboxylate O groups of Glycine, and two donor N atoms of 2-(1*H*-Imidazol-2-yl) pyridine (Impy) coordinate to copper in basal positions. A molecule of water occupies the apical position in the crystal. It is observed that Compound **2** interacts non-covalently with six hydrogen bonds (4OH⋯O, 2NH⋯O) with neighbors, as shown in Figure 4. Figure 5 shows how these hydrogen bonds are responsible for the supramolecular structure of the compound, as well as the π–π interactions (3.677 Å between centroids) help to stabilize the solid-state structure are presented. In Table 3, all the hydrogen bonds, including CH⋯O, are characterized. In Figure S2, a growing chain of metavanadate is depicted between two cationic units.

### 2.1. Visible Spectroscopy

The UV-Vis spectra of Compounds **1** and **2** are presented in Figures S3 and S4; respectively, both samples were dissolved in phosphate-buffered saline (PBS) solution at pH 7.4. The sample for Compound **1** was made at 2.2 mM, and it showed an absorption band at 668 nm, with an absorbance of 0.12; therefore, according to these data, the molar extinction coefficient is ε = 61 L mol^−1^ cm^−1^. The solution for Compound **2** was made at 1.6 mM, and it showed an absorption band at 635 nm, with an absorbance of 0.05. Thus, the molar extinction coefficient is ε = 57 L mol^−1^ cm^−1^. The main peaks at 669 and 635 nm are typical of transferring the electrons between the d–d orbitals of Cu (II) complexes [39,40].

### 2.2. FTIR Spectroscopy

The infrared spectrum of Compounds **1** and **2** are shown in Figures S5 and S6, respectively. Compound **1** shows a set of bands in the region from 3250 to 3150 cm^−1^ in the high-frequency region of the IR spectra and is attributed to the υs (N–H) stretching bands. The band corresponding to the symmetric vibration of methyl groups of HMetf can be seen at 2972 cm^−1^ and is of very weak intensity. Deformation vibrations of the NH bipyridine bonds were observed in the region of 1600–1500 cm^−1^ with strong intensity. The CN stretching vibrations were observed in the region of 1170 to 1040 cm^−1^, as medium intensity bands. A peak at 972 cm^−1^ is attributed to NH vibrations outside the plane. Bands between 580 and 418 cm^−1^ correspond to C-N-C [41,42], and Cu-N and Cu-O stretching vibrations.

In Figure S6, the band at 3084 cm^−1^ corresponds to the asymmetric stretching vibration of NH_2_. The band at 1034 cm^−1^ is attributed to stretching vibrations of the C-N and C-C bonds. However, it should be noted that the C-H vibration of the imidazole moiety could be in the same range that the VOterm symmetric and asymmetric vibrations (1300–1100 cm^−1^) [43]. Near 900–800 cm^−1^, the bridging V-O-V symmetric and antisymmetric stretching modes were observed. At 893 cm^−1^, there are twist vibrations of NH_2_ and CH_2_. The bands at 774, 756, and 650 cm^−1^ are attributed to the υs (VO-bridging), and the 607 and 417 cm^−1^ bands could be due to Cu-N or Cu-O vibrations of the coordinated aminoacidate ions [44].

### 2.3. Theoretical Calculations

Molecular structures of Compounds **1** [Cu(Metf)(bipy)(Cl)]^+^, **1′** [Cu(Metf)(bipy)(H_2_O)]^2+^, and **2** [Cu(Impy)(Gly)(H_2_O)]^+^ were calculated at the level of theory APFD/6-311+G(2d,p) using water as the solvent. Table 4 shows the relative electronic energies considering the ZPE correction ΔE_0_, the free energies of solvation, ΔG_sol_, and interaction energies, E_int_. The results of ΔE_0_ show that Compound **1** is the energetically most stable, with 384.0 a.u. less than Compound **1′** and 554.5 a.u. less than Compound **2**. The interaction between Cl and Cu confers high stability due to the electronegativity difference between them. The values of ΔG_sol_ show that the solvation of Compound **1′** is the most favored. A correspondence between ΔG_sol_ and E_int_ is observed. Compound **1′** presents the minor E_int_; this means that it is the more likely to carry out the solvation than Compounds **1** and **2**. For this hypothetical compound, the water molecule interacting with Cu atom presents a weaker interaction than Compounds **1** and **2**, respectively. In addition, Compound **1** has an E_int_ larger than Compound **2** by 140 kcal mol^−1^, approximately. However, they are only necessary 5 kcal mol^−1^ of ΔG_sol_ for carrying out the solvation between Compounds **1** and **2**. The values of E_int_ also show high stability arising from the interaction with the Cl^−^ ion in apical position respect to the axial positions in the square pyramidal geometry in Compound **1**. When the Cl^−^ ion is substituted by H_2_O, as in Compound **1′**, the value of E_int_ decreases considerably. The values of Compounds **1′** and **2** are similar, both containing H_2_O in the apical position in the square pyramid.

**Figure 6:**
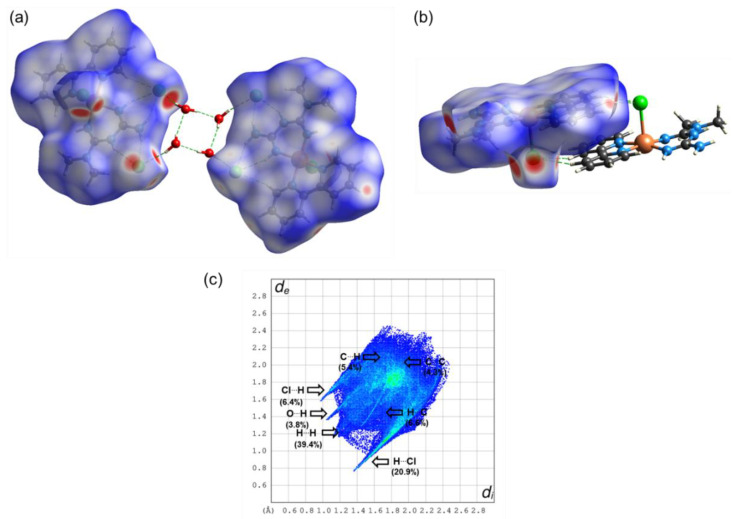
Hirshfeld surface mapped with d_norm_ parameter for a dimer of the Compound **1** showing (**a**) H and Cl bonds between molecules, (**b**) H bonds between molecules in π-stacking configuration, and (**c**) fingerprint plot of non-covalent interactions.

**Figure 7:**
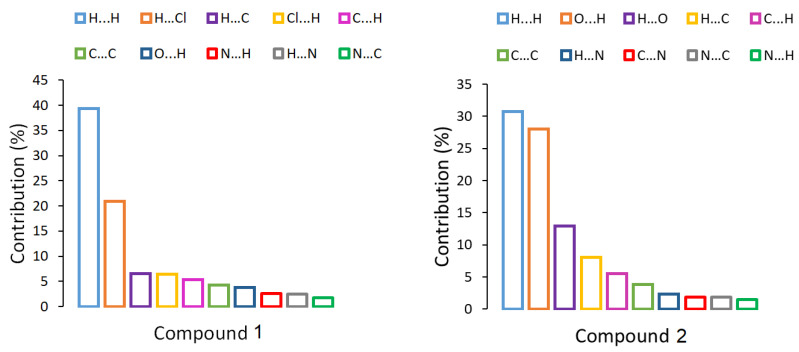
Percentage contributions to the Hirshfeld surface area for the main close intermolecular contacts of Compounds **1** and **2**.

**Figure 8:**
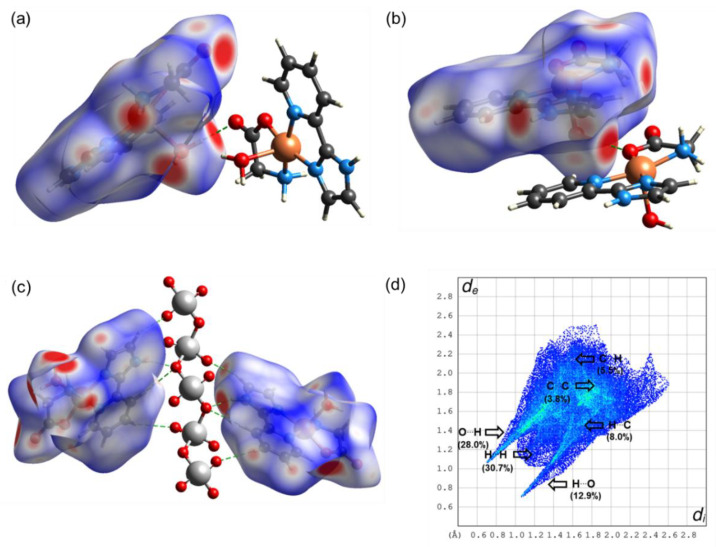
Hirshfeld surface mapped with d_norm_ parameter for a dimer of the Compound **2** showing (**a**) H bonds between molecules, (**b**) H bonds between molecules in π-stacking configuration, (**c**) H bonds between molecules with [(VO_3_)^−^]_n_ chain, and (**d**) fingerprint plot of non-covalent interactions.

The Hirshfeld surfaces were mapped with the normalized contact distance, d_norm_. Hirshfeld surfaces and a fingerprint plot of Compound **1** are shown in Figure 6. In Figure 6a, it is observed that the major red spots on the Hirshfeld surface are due to close intermolecular interactions between Cl⋯HO of the water molecules forming the cluster between two molecules. In addition, the non-covalent interactions between Cl and H of the bipy moiety of the adjacent molecules have a significant contribution, as is shown in Figure 6b. Strong hydrogen bonds O⋯H (i.e., 2.00 and 2.17 Å; 163.7 and 133.5°) are observed between the water molecules of the cyclic arrangement shown in Figure 3. The fingerprint plot in Figure 6c shows the main non-covalent interactions. d_i_ indicates the distance from the surface to the nearest nucleus inside the surface, and d_e_ is the distance from the surface to the nearest nucleus outside the surface. Figure 6c shows the typical fingerprint for the Cl⋯H halogen bond [45]. The most significant contributions are from interactions H⋯H (39.4%), H⋯Cl (20.9%), H⋯C (6.6%), and Cl⋯H (6.4%). Other interactions with minor contributions are H⋯C (5.4%), C⋯H (5.4%), and C⋯C (4.3%) (see Figure 7). For Compound **2**, Figure 8a shows the red spots on the Hirshfeld surface due to the main H-bond interaction between H of H_2_O coordinated with Cu, in the apical position of the square pyramidal geometry, with O of C=O group of Gly, when O acts as a donor. This interaction O⋯H is a strong H bond with an interatomic distance of 2.01 Å and valence angle OHO of 174.53°. The interaction H⋯O, when O acts as acceptor, shows the same red spot on the surface as in Figure 8b. It can be observed as a typical cyclic O⋯H hydrogen bond in the fingerprint plot [45] in Figure 8d. Red spots are also observed for interactions H⋯OV between H of Impy moiety and O of metavanadate [(VO_3_)^−^]_n_ chain, as shown in Figure 8c. In the fingerprint plot in Figure 8d, the most significant contributions are attributed to H⋯H (30.7%), O⋯H (28.0%), and H⋯O (12.9%). The cyclic hydrogen bond O⋯H shows an upper spike in the fingerprint plot associated with the donor oxygen atoms and the lower spike associated with the acceptor oxygen atoms. This cyclic hydrogen bond was also observed in a complex [DMAPH]_4_[H_2_V_10_O_28_]5H_2_O, where DMAPH is 4-dimethylaminopyridinium acting as counterions of the decavanadate anion, contributing highly to a supramolecular arrangement [46]. This interaction also arose from interactions between H atoms of organic counterions and O atoms of vanadate species. Other interactions with minor contributions are H⋯C (8.0%), C⋯H (5.5%), and C⋯C (3.8%), among others (see Figure 7).

**Figure 9:**
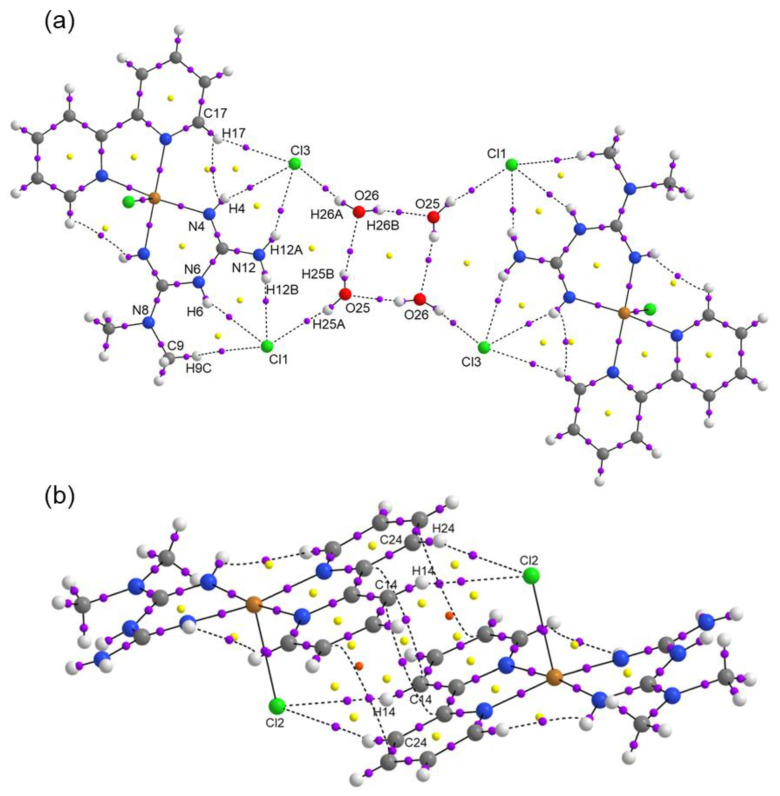
Molecular graphs for a dimer of the Compound **1** showing (**a**) H and Cl bonds between molecules, and (**b**) H bonds between molecules in π-stacking configuration.

**Figure 10:**
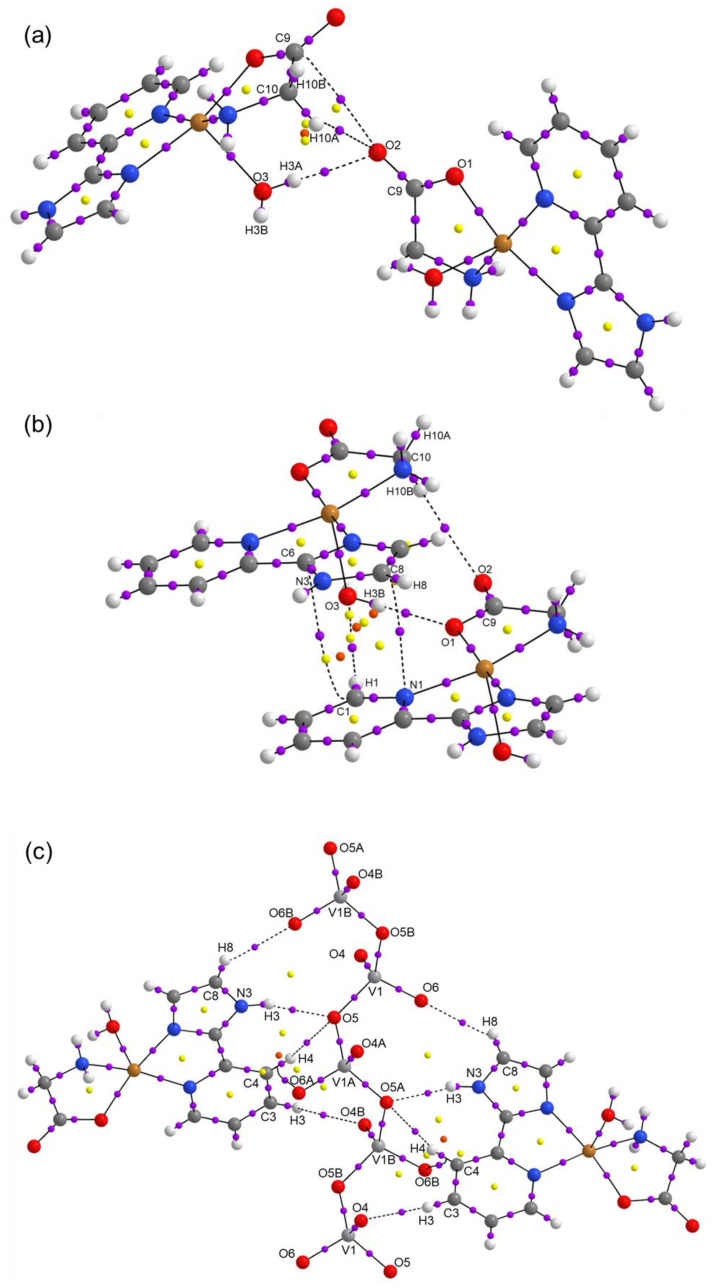
Molecular graphs for a supramolecular dimer of Compound **2** showing (**a**) H bonds between molecules, (**b**) H bonds between molecules in π-stacking configuration, (**c**) H bonds between molecules with [(VO_3_)^−^]_n_ chain.

Topological parameters used to characterize intramolecular interactions, such as electron density ρ(r), Laplacian $\nabla^{2}\rho \left(r\right)$, Lagrangian kinetic energy *G*, potential energy density *V*, Hamiltonian kinetic energy *H*, interaction energy *E_H…Y_*, and interatomic distance $D_{inter}\;$, are shown in Table 5. The value of the equation H(r)=G(r)−V(r) determines the molecular interaction regions, and interaction energy is calculated from the equation $E_{H\cdots Y}=\frac{1}{2}\left|V\left(r\right)\right|$ [47]. The molecular graphs for Compounds **1** and **2** are shown in Figure 9 and Figure 10, respectively. Purple dots represent the bond critical points (BCPs), yellow dots represent the ring critical points (RCPs), and orange dots represent the cage critical points (CCPs). Figure 9a presents the main H bond interactions between Cl atoms and H atoms of organic counterions (Metf and bipy) and water molecules. For Compound **1**, the ρ(r) for H bonds are in the range 0.0062–0.0213 a.u. The maxima ρ(r) were found for the intermolecular O25⋯H26_B_ and Cl3⋯H26_A_ (see Figure 9a) which contribute thoroughly to the crystal packing. Their *E_H…Y_* values indicate that they are the most stabilized interactions with 4.96 and 4.08 kcal mol^−1^. The interactions Cl2…H24 and Cl2…H14, do not have the highest values of ρ(r) Nevertheless, they play an essential role in the supramolecular structure. In Figure 9b, it can be seen that there are a lot of RCPs, indicating the formation of rings, and two CCPs form cage structures between the dimer of the molecules of Compound **1**. These RCPs and CCPs provide structural stability to the molecular packing shown in Figure 3. For Compound **2**, the ρ(r) for H bonds are in the range 0.0056–0.0194 a.u. The interactions O2⋯H10_A_ in Figure 10a, O1⋯H3_B_ in Figure 10b, and O5⋯H3 in Figure 10c have the maxima values of ρ(r). These interactions are the most stabilized with *E_H…Y_* of 4.61, 5.18, and 4.27 kcal mol^−1^, respectively. Similarly to Compound **1**, the interactions between the dimer of the molecules of Compound **2** are stabilized by the formation of RCPs and CCPs, as shown in Figure 10b. The interactions H⋯OV between H of Impy moiety and O of the metavanadate [(VO_3_)^−^]_n_ chain are fundamental for the stability of the molecular packing shown in Figure 5. In all cases, for Compounds **1** and **2**, the positive values of $\nabla^{2}\rho \left(r\right)$ confirm the hydrogen bond behavior of the interactions. Positive values of H(r) indicate hydrogen bonds of a purely electrostatic nature [48].

### 2.4. Molecular Docking (DNA)

In Table 6, the docked binding energies and the interaction with DNA corresponding to the top molecular poses (lowest energy) for Doxorubicin, Compound **1′** [Cu(Metf)(bipy)(H_2_O)]^2+^, Compound **2** [Cu(Impy)(Gly)(H_2_O)]^+^, and six other compounds with similar structures are presented.

**Figure 11:**
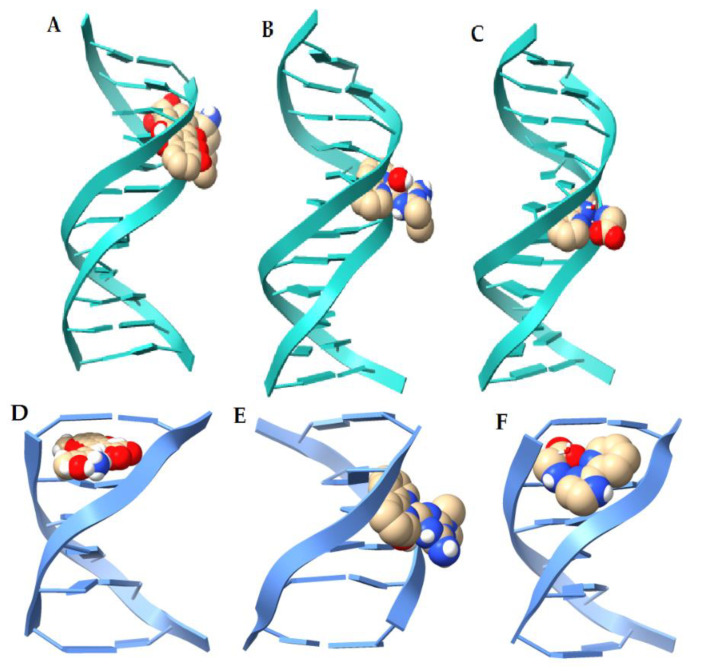
Docked structures of the top molecular poses for doxorubicin (**A**) and copper complexes (**B**,**C**) with DNA(1BNA) fragment; and doxorubicin (**D**) and copper complexes (**E**,**F**) with DNA (151D) fragment.

In Figure 11, the best poses for the interactions of Compounds **1** and **2**, and the molecule of doxorubicine with the DNA fragments 1BNA and 151D, respectively, are presented. Interestingly, all docked structures occupy equivalent positions in the minor groove of the 1BNA DNA fragment structure. However, the best affinity energies are found in the compounds doxorubicin, [Cu(bipy)(Orn)(H_2_O)]^2+^, [Cu(phen)(Lys)(H_2_O)]^2+^, [Cu(bipy)(Lys)(H_2_O)]^2+^, [Cu(phen)(Orn))(H_2_O)]^2+^. Thus, we can imply that ligands ornithine and lysine lead to favorable affinity energies by forming hydrogens bonds and salt bridges with the DNA structure. It is well known that doxorubicin intercalates into DNA [49]. In previous work [38,39], we have shown that our compounds also can intercalate into DNA. All compounds intercalate well with DNA structure, although compounds **1′**, **2**, **5**, and **8** have minor binding energies. In addition, our compounds seem to better interact with DNA by minor-groove binding. This type of interaction has also been reported in previous studies with Casiopeinas^®^ [50,51]. It is interesting to point out that the [Cu(Metf)(bipy)(H_2_O)]^2+^ prefers the minor groove binding in both DNA fragments.

**Table 6:** Docking results. Binding energies for the best molecular poses of the complexes between copper compounds, doxorubicin, and DNA fragments.

Ligand	Binding Energy (kcal/mol) 1BNA [52]	Binding Energy (kcal/mol) 151D [53]	Interaction
Doxorubicine	−11.09	−11.54	H bond, π-anion
1’ [Cu(Metf)(bipy)(H_2_O)]^2+^	−9.69	−7.05	H bond, salt-bridge
2. [Cu(Impy)(Gly)(H_2_O)]^+^	−8.82	−6.73	H bond, π-anion
3. [Cu(phen)(Lys)(H_2_O)]^2+^ [37]	−11.03	−9.98	H Bond, π-anion, salt-bridge
4. [Cu(bipy)(Orn)(H_2_O)]^2+^ [37]	−11.12	−9.68	H bond, salt-bridge, π-anion
5. [Cu(phen)(Gly)(H_2_O)]^+^ [38]	−9.5	−8.52	H bond,
6. [Cu(phen)(Orn))(H_2_O)]^2+^ [#]	−11.05	−9.43	H bond, salt-bridge, π-anion
7. [Cu(bipy)(Lys)(H_2_O)]^2+^ [#]	−11.04	−8.72	H bond, salt-bridge
8. [Cu(phen)_2_(H_2_O)]^+^ [54]	−8.79	−8.53	H bond, π-anion

# Unpublished results.

### 2.5. Molecular Docking (tRNA)

**Figure 12:**
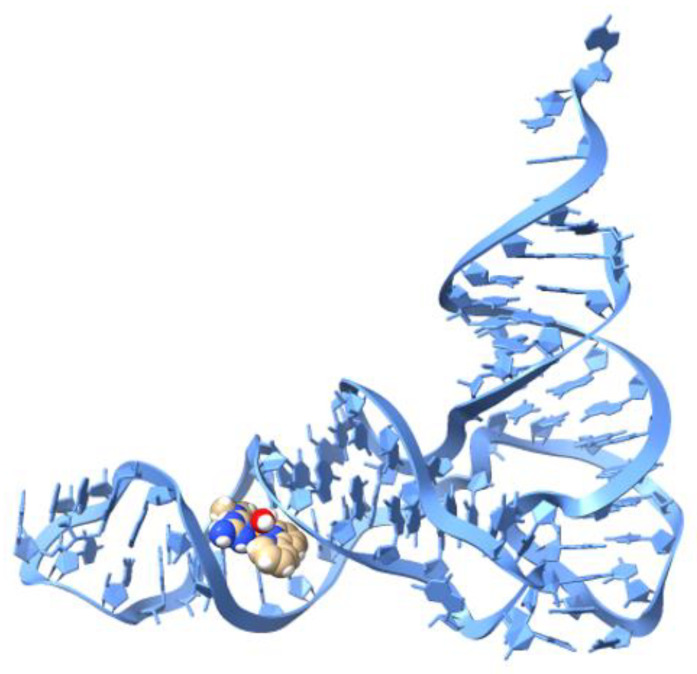
The best fit molecular docked pose of [Cu(Metf)(bipy)(H_2_O)]^2+^ complex with tRNA.

The transfer RNAs (tRNA) are small RNA molecules that translate the genetic code into amino acids. In recent years, tRNA has gained relevance due to its widespread deregulated expression in cancer cells [55]. To elucidate the capability of our compounds to interact with tRNA, we docked them with the yeast tRNA (PDB ID: 6TNA) [56]. Yeast tRNA is a well-defined 3D structure with different structural motifs that participate with targets for specific base-pair recognition. These structures are known as D arm, acceptor stem, T arm, ψ loop, and anticodon arm [57]. Additionally, to Compounds **1** and **2**, docking was performed with doxorubicin and [Cu(hydroxynaphthaldehyde)(H_2_O)] to compare them, since these molecules have been previously reported to have the ability to interact with tRNA [58,59]. The binding energies are presented in Table 7. From this table, it can be seen that among the four structures docked; Compound **1′** has the best binding energy interaction with tRNA (Figure 12), followed by Doxorubicin, Compound **2**, and [Cu(hydroxynaphthaldehyde)(H_2_O)]. These compounds share similar binding sites into the anticodon arm on tRNA in proximity to G-24, C-25, A-26, C-27, A-39, C-40, U-41, G-42. The interactions involved several H-bond and hydrophobic interactions. It is important to highlight that the free NH_2_ group present in Compounds **1′**, **2**, and DOX seems to have an important role in the drug–tRNA interaction [59]. 

## 3. Discussion

Copper complexes are among the most studied transition metal complexes for their antitumor properties because endogenous metal ions may lead to less systemic toxicity. The properties of the copper complexes are determined mainly by the nature of their ligands, which themselves may exhibit antiproliferative activity [60]. Hence, the ligands surrounding the metal ion are of extreme importance, since they can neutralize the electrical charge of the copper ion and facilitate the transport of the complex through the cell membrane. Ligands can also help the copper complexes to interact non-covalently with proteins or to intercalate into DNA strands [61], and also induce DNA damage through hydrolytic or oxidative cleavages [62,63]. The biological activity displayed by copper-based complexes of similar structures is plentiful, and several applications have been found. Zoroddu et al. (1996) reported a significant activity of copper–phenantroline complexes against Gram-positive and Gram-negative bacteria [64]. Patel et al. (2005) showed that the copper(II) complex with L-Phenylalanine exhibited substantial activity against some human pathogens, particularly against *Bacillus subtilis*, and a significant antifungal activity against *Aspergillus terreus*. Thus, it may be concluded that copper(II) complexes inhibit the growth of bacteria and fungi to a greater extent [65]. Also, the growth inhibition of the *Giardia lamblia* parasite [66] and the antiparasitic activity against *Trypanosoma cruzii* have been reported for ternary complexes based on bipyridine [67]. Copper complexes incorporating Schiff bases, amino acids, peptides, azoles, terpyridines, or polypyridyls as ligands as well as dinuclear copper complexes and copper complexes incorporating natural products or bioactive ligands showing metallonuclease activity have been reviewed [68]. Recently, a series of ternary copper(II)-L-dipeptide-neocuproine complexes have shown cytotoxicity against cancer cells, including MDA-MB-231, triple-negative breast cancer [69]. The complex bis[(μ2-chloro)chloro(1,10-phenanthroline)copper(II)] exhibited a potent anticancer activity against B16, MDA-MB-32, A549, HT-29 and SF, cell lines, with an average IC_50_ value of 0.726 μg/mL (1.15 μM), compared to 4.88 μg/mL (16.3 μM) for cisplatin. Additionally, it showed a better selectivity against cancer cells compared to human bone marrow stem cells and less toxicity on rats as compared to cisplatin. The anti-cancer activity of complex could be attributed to the adequate delivery of copper to tumor cells with the aid of phenanthroline, leading to the inhibition of proteasome and elevation of intracellular oxidative damage [70]. Copper(II) piperazine- and piperidine-based dithiocarbamates that exhibited distorted square planar geometry have shown promising biological potential, as evidenced by DNA-binding, antileishmanial, antioxidant, and brine shrimp cytotoxicity [71]. Copper(II) complexes of metronidazole and 1,10 phenanthroline were found to demonstrate potential antimicrobial and antifungal properties. Theoretical studies indicated that key amino acid of the active sites of *C. albicans* (CYP51), *K. pneumoniae* (4HL2), and *E. faecium* (4M7U) interacts with electron-donor and electron-withdrawing substituents of copper(II) complexes. These compounds could be used as possible lead compounds for the design of more potent antimicrobial and antifungal agents. [72] [Cu(py-phen)(asn))(H_2_O)]NO_3_ and [Cu(py-phen)(trp)(H_2_O)]NO_3_ (py-phen: pyrazino[2,3-f][1,10]phenanthroline, asn: asparagine, trp: tryptophan) were recently reported [73]. The complexes have shown radical scavenging activity and anti-cancer activities against three cancer cell lines (MCF-7, Caco-2, and A549) and a non-tumor cell line (BEAS-2B). Recently, the review “Copper Coordination Compounds as Biologically Active Agents” concluded that the redox activity of copper ions along with their biogenicity, the stability of copper coordination compounds in the bloodstream, and the highly promising therapeutic results in vitro and in vivo prove the potential of copper coordination compounds to become widely used in clinical practice [74].

The molecular structure of Compounds **1** and **2** are highly reminiscent of Casiopeinas^®^ (CAS), which are a series of copper-based drugs developed by Ruiz-Azuara and coworkers [75,76]. CAS are mixed chelate copper(II) complexes with the general condensed formula [Cu(N–N)(A–A)]NO_3_, where N–N represents neutral diimmine donors, either phenantroline or bipyridine derivatives, and A–A stands for uni-negative N–O or O–O donors, either aminoacidates or acetylacetonate [77,78]. CAS were designed as a chemotherapeutic alternative for cancer treatments and according to some preliminary experiments, some of them have shown antineoplastic activity both in vitro and in vivo, and currently, they are in phase I clinical trials in Mexico [79]. As shown above, DNA is the primary target molecule for most anti-cancer and antiviral therapies. Therefore, our goal has been to develop planar organic compounds that can bind to DNA by intercalating aromatic heterocyclic rings such as phenanthroline/bipyridine between the DNA base pairs. In this work, two new compounds are shown that can interact with DNA as groove binders and moderated intercalators. However, the docking results with tRNA are more promising, since in a recent article, the ability of [Cu(hydroxynaphthaldehyde)(H_2_O)] to act as a metallonuclease was demonstrated [56]. The complex exhibited selectively remarkably good cytotoxic potential on leukemia (K-562), cervical (HeLa), and hepatoma (Hep-G2) cancer cell lines.

Besides the pure inorganic point of view, they provide interesting solid-state supramolecular structures with various non-covalent interactions, which requires further investigation, especially from the magnetic point of view.

Our group has previously synthesized Cu/V heterobimetallic compounds; therefore, our aim was to complete a series of such complexes containing a planar copper center and vanadium in the form of cyclo-tetravanadate. However, while we followed similar synthetic procedures as before, subtle changes in the reaction pot prevented us from obtaining such complexes. Compound **1** contains only copper, and Compound **2**, although bimetallic, contains vanadium as the reagent vanadium species metavanadate. This behavior can be explained because of the stability of the compounds in the solid-state is mainly due to extensive non-covalent interactions. For Compound **1**, the abundance of chlorine atoms during the reaction might be responsible for the obtained structure that involves a cluster of four water molecules and chlorine atoms with seven neighboring contacts, as well as the peculiar supramolecular structure. In addition, the metformin molecule was found with a neutral charge. The cyclic water cluster is classified as a type A tetramer, according to Cobar et al. [80].

There are four donor-acceptor interactions, with the distances of 2.000 and 2.169 Å, and the angles 163.68° and 133.47°. The interactions of chlorine atoms with water molecules at distances of 2.505 and 2.279 Å and the angles 168.85° and 170.12° were also characterized as significant non-covalent interactions in the solid-state molecular packing. For Compound **2**, interesting arrangements involving non-covalent interactions, which dictate their supramolecular structures, were found. The strong H bond interactions with a distance of 2.01 Å and valence angle of 174.53° are responsible for the arrangement in the dimer form of Compound **2**. Vanadium in the form of a metavanadate chain interacts through hydrogen bonds with the copper cationic moieties, which also make a supramolecular structure via hydrogen bonds and π–π interactions. A comparison with the reported structures of metavanadates of sodium, ammonium, and potassium is worthwhile since the hydrogen bond interaction with the neighbors is not present in the salts of sodium and potassium. However, they are present in the ammonium salt. The distances of V1-O5 and V1-51 are practically the same and are involved in constructing the metavanadate chain. The other two distances are shorter and different since they interact with a different set of hydrogen bonds. In the Supplementary Material section, Table S7 shows the similarity with the ammonium salt, although the hydrogen bonds are with the same neighbors. However, in that case, it also shows small differences [81,82,83]. This compound shows a characteristic cyclic hydrogen bond, which also has been observed in a complex of decavanadate with 4-dimethylaminopyridinium acting as their counterions [46]. This interaction contributes highly to the supramolecular arrangement due to the formation of cyclic arrangements stabilizing the whole structure. The formation of cyclic arrangements through O…H bonds also has been evaluated using AIM analysis founding values of ρ(r) in the BCP of 0.0211 a.u. and RCP of 0.0020 a.u. [46]. In Compounds **1** and **2**, values of ρ(r) of 0.0213 and 0.0194 a.u. for BCP in more relevant hydrogen bonds were found, corresponding to the highest interaction energies, E_int_. In Compound **1,** a value of ρ(r) in the RCP of 0.0104 a.u. was found for the cyclic water cluster. The Hirshfeld surface and AIM analyses have supported the relevance of these non-covalent interactions in stabilizing the molecular packing and supramolecular characteristics of this kind of compound. Hydrogen bond and halogen bond interactions have been adequately characterized by the Hirshfeld surface and AIM analyses, showing these non-covalent interactions contribute to the supramolecular characteristics of the Compounds **1** and **2**, with high contribution percent of the Hirshfeld surface and with high interaction energies. Hydrogen and halogen bonds are an attractive interaction where the electrophilic atom approaches a negatively polarized species, which provides unique characteristics in the strength, size, and interaction gradation to play a recurring role in forming supramolecular structures from structural units [84]. In complexes containing vanadate species, hydrogen bonds, halogen bonds, or the combination of them have been studied for their importance in the formation of diverse supramolecular patterns. Intermolecular interactions in different decavanadate complexes with cations and solvent molecules were analyzed, finding that they characterize the assembly patterns present in the crystal structure [85]. In polyoxovanadate clusters stabilized by diverse cations and containing transition metals such as Co and Zn have been found crystal patterns due to water–water hydrogen bonding and interactions among the lattice water, mainly cyclic pentamers, and zinc coordinated water molecules [86].

## 4. Materials and Methods

Ammonium metavanadate, 2,2′-bipyridine (bipy), glycine monohydrochloride (Gly), and 2-(1H-Imidazol-2-yl) pyridine (Impy) were purchased from Sigma-Aldrich. KOH was purchased from Fermont, and CuCl_2_·2H_2_O was purchased from Química Dinámica S. A. de C. V. (Monterrey, Mexico). Metformin hydrochloride (Metf) was obtained by extraction of commercial over-the-counter tablets.

All manipulations were carried out at room temperature with no special solvent and reagents purification. Elemental analysis (EA) was carried out on a Perkin Elmer 2400 Series II CHNO/O Analyzer. The electronic spectra of the complexes were determined by UV-Vis spectroscopy with a Varian Cary 50 spectrophotometer with a xenon lamp and using a quartz cuvette of 1 cm path length. Infrared spectra were obtained in KBr pellets from 400 to 4000 cm^−1^ using an IR Digilab, Mod. Scimitar FTIR spectrophotometer. Single-crystal X-ray data were recorded with an Agilent Gemini A diffractometer and data were refined using the SHELX-2014/7 software [87]. Selected crystal data and details of the structure determination of the compounds are shown in Table 1 and Tables S1–S6. The CCDC numbers are 2024492 (Compound **1**) and 2024712 (Compound **2**). In the supplementary section, additional crystallographic data for this paper are presented. Complete data can be obtained free of charge at http://www.ccdc.cam.ac.uk/conts/retrieving.html (or from the CCDC, 12 Union Road, Cambridge CB2 1EZ, UK; Fax: +44-1223-336-033; e-mail address: deposit@ccdc.cam.ac.uk). The crystal structures were studied using Mercury CSD (release 4.3.1) [88], which, together with OLEX-2, was used to produce crystallographic artwork [89]. 

### 4.1. Synthesis

Compound **1** was synthesized by mixing 0.001 mol (0.156 g) of 2,2′-bipyridine in 30 mL of distilled water with stirring and heat. After that, 0.001 mol (0.170 g) of CuCl_2_·2H_2_O were added and the mixture was cooled to room temperature. Later the pH of the solution was adjusted to 9.0 with the addition of 10% NaOH solution. Two tablets of metformin hydrochloride (850 mg each), previously crushed, were added to the prepared solution. A second solution containing 0.001 mol (0.116 g) of NH_4_VO_3_ in 15 mL of distilled water was prepared and added drop-wise to the copper(II) solution. The final mixture was filtered and left at room temperature; two weeks later, blue prismatic crystals were obtained, which were highly soluble in water. 

Compound **2** was prepared by the addition of 0.001 mol (0.145 g) of 2-(1*H*-Imidazol-2-yl) pyridine (Impy) in 30 mL of distilled water with stirring and heat; then 0.001 mol (0.075 g) of glycine (Gly) and 0.001 mol (0.170 g) of CuCl_2_·2H_2_O were added and mixed, and the solution was cooled to room temperature. Later the mixture was adjusted to pH 9.0 with NaOH (10%). Another solution was prepared to contain 0.001 mol (0.116 g) of NH_4_VO_3_ in 15 mL of distilled water, and it was added to the previous solution. The ultimate solution was filtered, and it was left at room temperature, getting blue crystals after one day.

### 4.2. Computational Methods

Geometry optimization of Compounds **1** and **2** was obtained using the functional APFD [90], and the triple zeta split-valence 6-311+G(2d,p) basis set [91]. Vibrational frequencies calculations were performed for identifying the stationary points on the potential energy surface. Implicit solvation was included with the Polarizable Continuum Model (PCM), using the integral equation formalism variant (IEFPCM) with water as the solvent [92]. Relative and interaction energies were computed, applying counterpoise correction [93]. Calculations were performed with the Gaussian16 program [94]. Non-covalent interactions were analyzed from X-ray structures using the Hirshfeld surface, and the 2D-fingerprint plot generated using CrystalExplorer 17.5 [95] and atoms in molecules analysis (AIM) using AIMAll software (Version 14.11.23, TK Gristmill Software, Cambridge, MA, USA) [96]. 

### 4.3. Molecular Docking

It was considered that in aqueous solution and because of reduction, reoxidation, and aquation, the copper compounds were studied as the aquo complexes. Molecular docking analysis was performed with the semi-flexible method. The DNA fragments used were Dickerson–Drew dodecamer (DDD) with the sequence d(CGCGAATTCGCG) 2 (PDB ID: 1BNA [52]) and DNA fragment with the sequence d(CGATCG) (PDB ID:151D [53]). The RNA docking was carried out using the yeast tRNA (PDB: 6TNA) [56] and was considered as a rigid entity. Complete flexibility was allowed for the coordination compounds [97]. Nine different compounds were considered comparing these two new compounds with the compounds previously reported by our lab and those that have been reported, and also, the well-known chemotherapeutic drug doxorubicin. The preparation of the macromolecule and the eight coordination compounds and doxorubicin, Compound **1′** [Cu(Metf)(bipy)(H_2_O)]^2+^, and Compound **2** [Cu(Impy)(Gly)(H_2_O)]^+^, was performed using the Autodock Tools 1.5.6 software [98] which includes the addition of polar hydrogens and empirical particles of atomic charges (Gasteiger-Marsili method). A grid box that encloses the entire DNA fragment was used with sizes 70, 70, and 120 Å for the 1BNA DNA fragment and 60, 60, and 76 Å for the 151D DNA fragment. For tRNA, blind docking was carried out using three different boxes that enclosed the entire tRNA with the sizes 96, 80, 108 Å, followed by a re-docking of the compounds in their docked pose with potential and preferred minimized energy using a box centered of the compounds with the sizes 40, 40, and 40 Å. The grid spacing for all the docking calculations was set to the 0.375 Å default value, using the Lamarckian genetic algorithm (LGA) searching methods. The parameters for the copper atom were the sum of VDW radii of two similar atoms (3.50 Å), plus the VDW well depth (0.005 kcal/mol), plus the atomic solvation volume (12.0 Å^3^), plus the atomic solvation parameter (−0.00110). The H-bond radius of the heteroatom in contact with hydrogen (0.0 Å), the well depth of the H-bond (0.0 kcal/mol) and different integers show the type of H-bonding atom and indexes for the generation of the auto grid map (0, −1, −1, 1, respectively). The corresponding figures were prepared using Chimera 1.14 [99] and Chimera X [100]. 

## 5. Conclusions

Two complexes were synthesized, one with Cu and another Cu/V. Experimental characterization was performed by visible and FTIR spectroscopies and with X-ray diffraction. Metformin, glycine, and bipyridine molecules were used as binders to form coordination compounds with copper. Although we were trying to complete a series of heterobimetallic vanadium/copper complexes, in this case, even though we follow the same reported procedure [37,38], the resulting compounds do not contain the cyclo-tetravanadate moiety. For Compound **1,** the vanadium component was absent, and for Compound **2,** it remains as the starting material metavanadate. This behavior can be explained because of the stability of the compounds in the solid-state, mainly due to extensive non-covalent interactions. In the first case, a cluster of four water molecules and chlorine atoms with seven neighboring contacts, and a peculiar supramolecular structure, was found. Additionally, the metformin molecule has a neutral charge. Interesting arrangements with non-covalent interactions dictating their supramolecular structures were found. In the second case, vanadium in the form of metavanadate interacts through hydrogen bonds with the copper cationic moieties, making a supramolecular structure via hydrogen bonds and π–π interactions. The Hirshfeld surface and AIM analyses supported the relevance of these non-covalent interactions in stabilizing the molecular packing of Compounds **1** and **2**. Hirshfeld surfaces analysis indicated the major zones prone to hydrogen and halogen bonds. From fingerprint plots, it was possible to quantify the contribution percent of these non-covalent interactions. From AIM analysis, different hydrogen and halogen bonds were characterized as strong non-covalent interactions from values of topological parameters based on electron density. 

The compounds can act as minor groove binders and relatively moderate intercalators for DNA and RNA molecules. Thus, opening potential applications as nanodelivery particles, polymeric micelles, nanoformulations, including supramolecular self-assembled structures, and bioconjugates are developed as part of the new strategies towards effective delivery of metallopharmaceuticals. Due to their interesting composition, structures, and potential interaction with DNA/RNA, further work on the evaluation of their cytotoxic activity is suggested in order to test their performance as potentially useful anti-cancer agents.

## Figures and Tables

**Table 1:** Crystallographic data.

	Compound 1	Compound 2
Empirical formula	C_14_H_23_Cl_2_CuN_7_O_2_	C_10_H_13_CuN_4_O_6_V
Formula weight	453.83	399.72
Temperature/K	293 (2)	293 (2)
Crystal system	Triclinic	Monoclinic
Space group	P-1	P2_1_/c
a/Å	8.4235 (3)	13.3902 (4)
b/Å	10.8688 (5)	5.21481 (14)
c/Å	11.2005 (4)	20.6731 (5)
α/°	108.249 (4)	90
β/°	93.220 (3)	107.316 (3)
γ/°	90.608 (3)	90
Volume/Å^3^	971.91 (7)	1378.13 (7)
Z	2	4
_δcalc_ g/cm^3^	1.551	1.927
μ/mm^−1^	1.421	2.257
F(000)	468	804
Crystal size/mm^3^	0.67 × 0.275 × 0.12	0.34 × 0.211 × 0.093
Radiation	Mo Kα (λ = 0.71073 Å)	Mo Kα (λ = 0.71073 Å)
2Θ range for data collection/°	5.99 to 77.408	5.918 to 70.408
Index ranges	−13 ≤ h ≤ 13, −17 ≤ k ≤ 17, −17 ≤ l ≤ 17	−20 ≤ h ≤ 21, −8 ≤ k ≤ 8, −33 ≤ l ≤ 33
Reflections collected	40,564	30,742
Independent reflections	8328 [R_int_ = 0.0940, R_sigma_ = 0.0687]	5883 [R_int_ = 0.0415, R_sigma_ = 0.0357]
Data/restraints/parameters	8328/0/247	5883/4/211
Goodness-of-fit on F^2^	1.009	1.031
Final R indexes [I ≥ 2σ (I)]	R_1_ = 0.0579, wR_2_ = 0.1322	R_1_ = 0.0405, wR_2_ = 0.0837
Final R indexes [all data]	R_1_ = 0.1070, wR_2_ = 0.1659	R_1_ = 0.0659, wR_2_ = 0.0962
Largest diff. peak/hole/e Å^−3^	0.58/−0.59	0.57/−0.85

**Table 2:** Hydrogen bond distances of the cyclic motif of Compound **1**.

D-H···A	D-H	H···A	D···A	D-H···A
O25-H25_B_···O26	0.851	2.169	2.822	133.47
O26-H26_B_···O25	0.850	2.002	2.828	163.68

**Table 3:** Hydrogen bond distances of Compound **2**.

D-H···A	D-H	H···A	D···A	D-H···A
NH···OVO_2_- (N3H3···O5)	0.861	2.050	2.877	160.96
HOH··· OCO- (O3H3_A_···O2)	0.755	2.019	2.771	174.53
HNH···OVO_2_- (N4H4_B_···O6)	0.788	2.196	2.914	151.77
HOH····OCO coord (O3H3_B_···O1)	0.773	1.995	2.762	171.17
CH····OCO- (C3H3_C_···O2)	0.930	2.569	3.436	155.30
CH····OH_2_ (C1H1···O3)	0.930	2.580	3.426	150.50
CH_2_····OCO- (C10H10_A_···O2)	0.970	2.587	3.406	142.16
CH····OVO_2_- (C3H3_C_···O6)	0.930	2.541	3.139	122.36
CH····OVO_2_- (C4H4····O5)	0.931	2.653	3.506	152.71
CH····OVO_2_- (C8H8···O4)	0.930	2.431	3.101	128.85

**Table 4:** Relative electronic energies with ZPE correction (ΔE_0_) (a.u.), relative free energies of solvation (ΔG_sol_), and interaction energies (E_int_) (in kcal mol^−1^) for Compounds **1**, **1′** and **2**.

Compound	ΔE_0_ (a.u.)	ΔG_sol_ (kcal mol^−1^)	E_int_ (kcal mol^−1^)
**1**	0.00	−67.58	−152.87
**1′**	384.03	−137.26	−11.33
**2**	554.52	−62.49	−12.50

**Table 5:** Topological parameters (a.u.), interaction energies E_H…Y_ (kcal mol^−1^), and interatomic distances D_int_ (Å).

BCP	ρ(r)	∇^2^ρ(r)	G (r)	V (r)	H (r)	E_H…Y_	D_inter_
**Compound 1**							
Cl3⋯H17	0.0098	0.0031	0.0062	−0.0046	0.0108	1.44	2.279
Cl3⋯H4	0.0094	0.0325	0.0064	−0.0046	0.0110	1.44	2.699
Cl3⋯H12_A_	0.0133	0.0469	0.0093	−0.0069	0.0162	2.16	2.504
Cl1⋯H12_B_	0.0189	0.0661	0.0139	−0.0114	0.0253	3.58	2.334
Cl1⋯H6	0.0113	0.0379	0.0075	−0.0056	0.0131	1.76	2.603
Cl1⋯H9_C_	0.0072	0.0217	0.0044	−0.0034	0.0078	1.07	2.933
Cl3⋯H26_A_	0.0204	0.0686	0.0151	−0.0130	0.0281	4.08	2.279
O25⋯H26_B_	0.0213	0.0885	0.0189	−0.0158	0.0347	4.96	2.002
Cl1⋯H25_A_	0.0126	0.0428	0.0085	−0.0062	0.0147	1.95	2.505
O26⋯H25_B_	0.0166	0.0644	0.0140	−0.0119	0.0259	3.73	2.168
Cl2⋯H24	0.0062	0.0177	0.0036	−0.0028	0.0064	0.88	2.951
Cl2⋯H14	0.0094	0.0290	0.0058	−0.0044	0.0102	1.38	2.724
**Compound 2**							
O2⋯H3_A_	0.0056	0.0204	0.0043	−0.0034	0.0077	1.07	2.019
O2⋯H10_A_	0.0194	0.0906	0.0187	−0.0147	0.0334	4.61	2.767
O6_B_⋯H8	0.0096	0.0362	0.0075	−0.0060	0.0135	1.88	2.431
O2⋯H10_B_	0.0071	0.0232	0.0050	−0.0042	0.0092	1.32	2.587
O1⋯H3_B_	0.0211	0.0966	0.0203	−0.0165	0.0368	5.18	1.995
O5⋯H3	0.0193	0.0794	0.0167	−0.0136	0.0303	4.27	2.049
O5⋯H4	0.0064	0.0211	0.0045	−0.0038	0.0083	1.19	2.653
O4_B_⋯H3	0.0083	0.0304	0.0064	−0.0052	0.0116	1.63	2.541

**Table 7:** Docking results. Binding energies for the best molecular poses of the complexes between copper compounds, doxorubicin, and tRNA.

Ligand	Binding Energy (kcal/mol) 6TNA	Interaction
Doxorubicin	−9.82	H bond, van der Waals, π-anion
[Cu(hydroxynaphthaldehyde)(H_2_O)]	−7.98	H bond, van der Waals, π-anion, π-π
1′ [Cu(Metf)(bipy)(H_2_O)]^2+^	−12.76	H bond, van der Waals, π-anion
2 [Cu(Impy)(Gly)(H_2_O)]^+^	−8.86	H bond, van der Waals, π-anion

## Supplementary Materials

The following are available online at. Two structure related figures, two Visible spectra, and two Infrared spectra complement the information given in the article. As well as seven tables containing structural information are available online, Figures S1–S6; Tables S1–S7.
