# Supplementary material for: Synthesis, Crystal Structure, and Computational Methods of Vanadium and Copper Compounds as Potential Drugs for Cancer Treatment
Source: Molecules. 2020 Oct 14;25(20):4679. doi: 10.3390/molecules25204679 (PMC7587343; doi:10.3390/molecules25204679)
Supplement: Supplementary file 1 [file molecules-25-04679-s001.pdf]

# Supplementary Material

**Nidia D. Corona-Motolinia[1], Beatriz Martínez-Valencia[1], Lisset Noriega[2], Brenda L. Sánchez-Gaytán[1], Miguel Ángel Méndez-Rojas[3], Francisco J. Meléndez-Bustamante[2], María Eugenia Castro[1*], and Enrique González-Vergara[1*]**

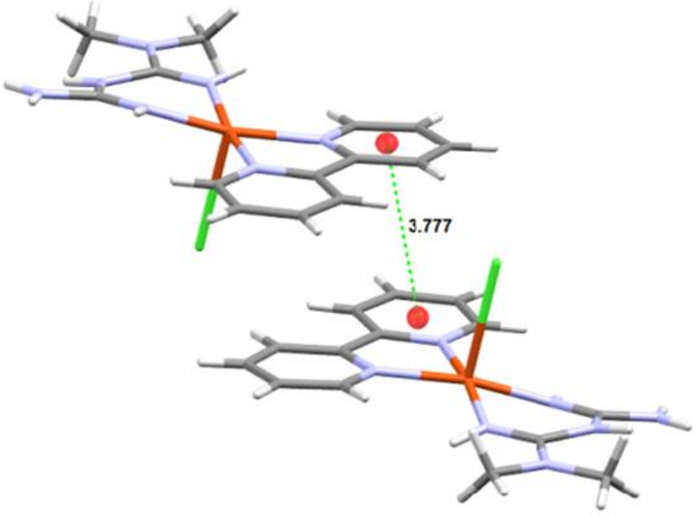


**Figure S1.** Capped stick representation of **Compound 1,** showing the distance between centroids of adjacent rings of bipyridine molecules.

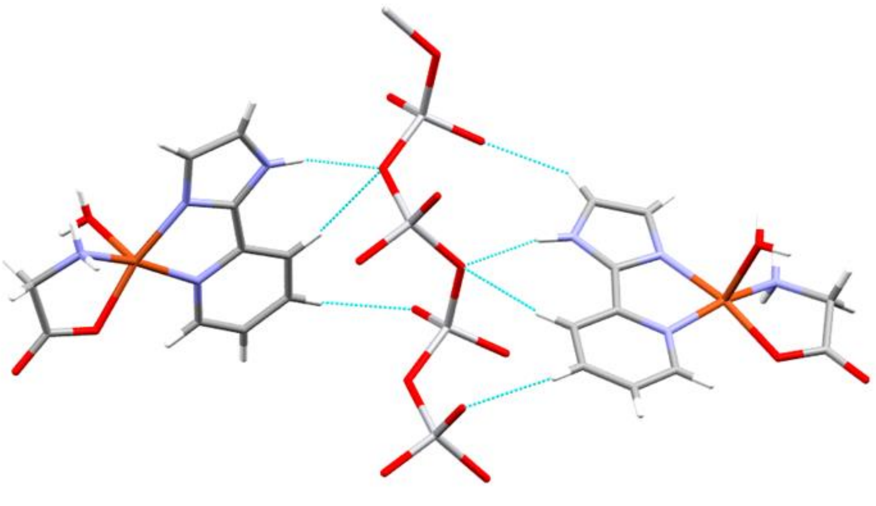

**Figure S2**. Capped stick representation of the hydrogen bond interactions of a growing metavanadate chain between layers of **Compound 2.**

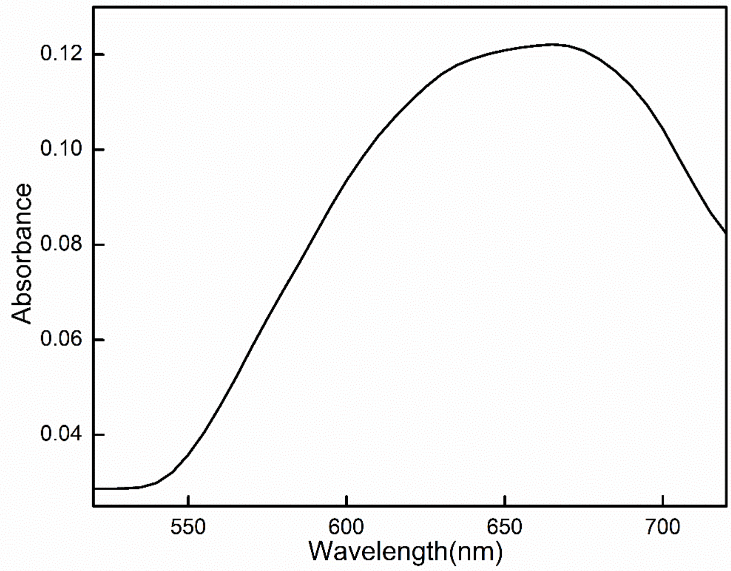


**Figure S3.** Visible spectrum of **Compound 1.**

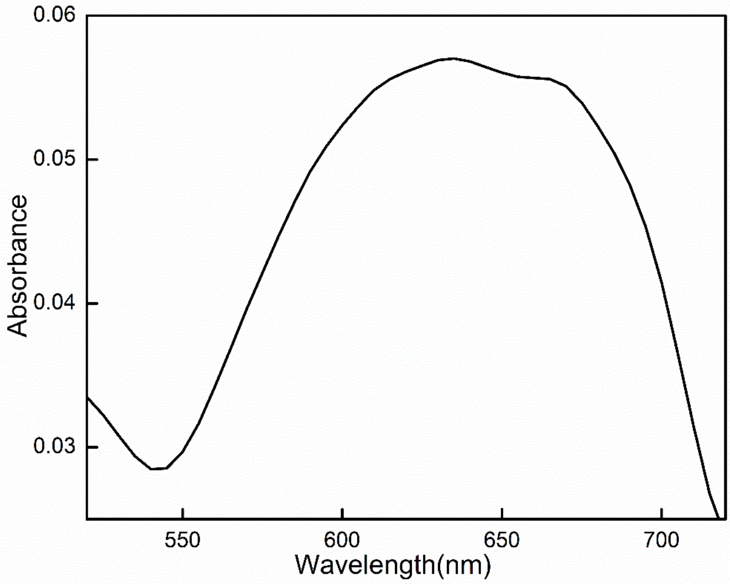


**Figure S4**. Visible Spectrum of **Compound 2**.

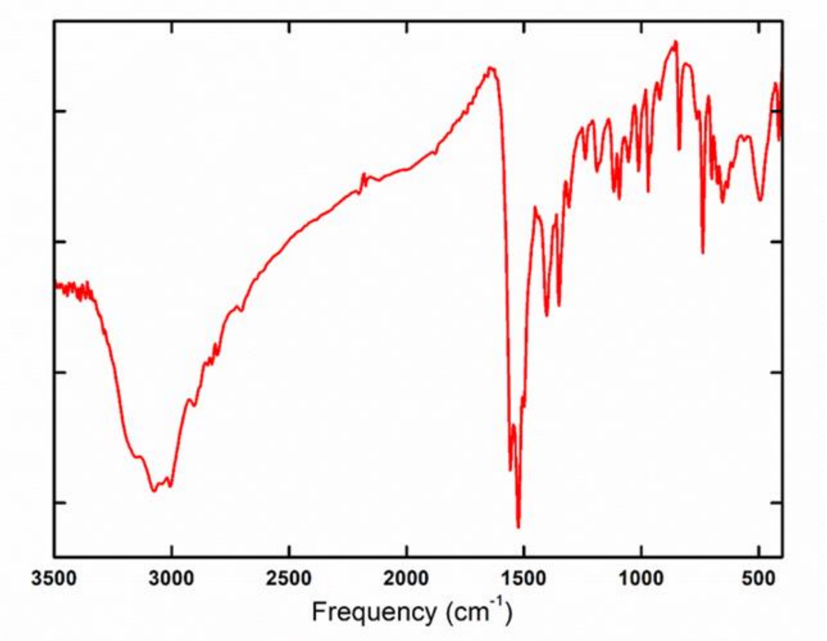


**Figure S5**. The infrared spectrum of **Compound 1**.

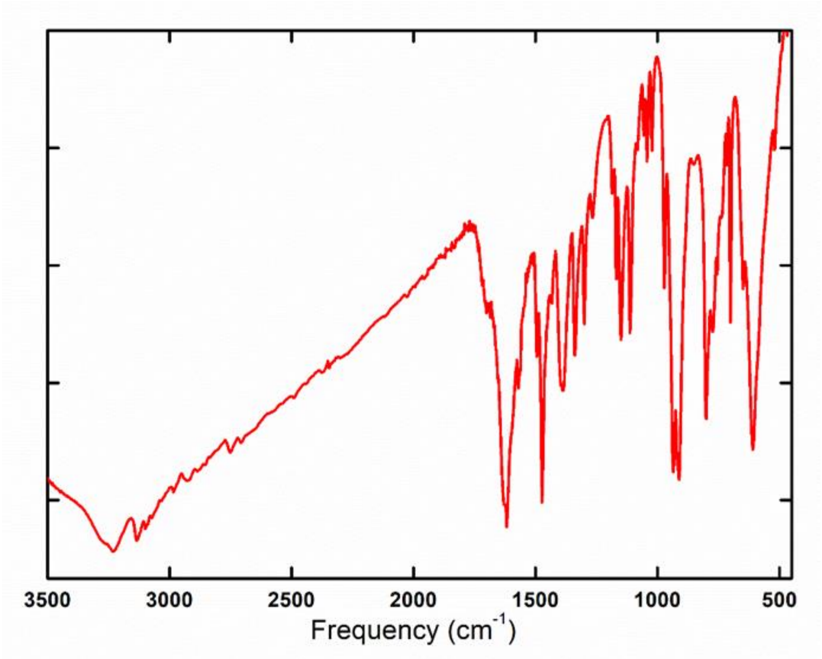


**Figure S6.** The infrared spectrum of **Compound 2.**

**Table S1**. Fractional atomic coordinates and isotropic or equivalent isotropic displacement parameters ($Å^2$) for **Compound 1.**

| | X | y | z | Uiso*/Ueq |
|---|---|---|---|---|
| Cu1 | 0.60984 (3) | 0.33069 (3) | 0.45565 (2) | 0.03412 (10) |
| Cl2 | 0.87935 (8) | 0.19152 (7) | 0.46484 (7) | 0.04906 (17) |
| Cl3 | 1.31805 (9) | 0.36561 (7) | 0.10889 (6) | 0.05270 (19) |
| N8 | 0.8329 (3) | 0.5067 (2) | 0.22254 (19) | 0.0415 (5) |
| N6 | 0.8503 (2) | 0.5601 (2) | 0.44002 (18) | 0.0345 (4) |
| H6 | 0.934711 | 0.604433 | 0.438060 | 0.041* |
| N4 | 0.6887 (3) | 0.4954 (2) | 0.5736 (2) | 0.0390 (5) |
| N18 | 0.5119 (2) | 0.2687 (2) | 0.58910 (18) | 0.0353 (4) |
| N11 | 0.6729 (3) | 0.3966 (2) | 0.32120 (19) | 0.0405 (5) |
| H11 | 0.621437 | 0.363299 | 0.249466 | 0.049* |
| N12 | 0.8668 (3) | 0.6694 (2) | 0.6516 (2) | 0.0431 (5) |
| H12A | 0.839717 | 0.683245 | 0.727516 | 0.052* |
| H12B | 0.938672 | 0.718067 | 0.636080 | 0.052* |
| N20 | 0.4788 (2) | 0.1737 (2) | 0.34283 (19) | 0.0356 (4) |
| C5 | 0.7971 (3) | 0.5723 (2) | 0.5578 (2) | 0.0305 (4) |
| C7 | 0.7815 (3) | 0.4838 (2) | 0.3250 (2) | 0.0322 (4) |
| C13 | 0.4043 (3) | 0.1693 (2) | 0.5459 (2) | 0.0352 (5) |
| C19 | 0.3841 (3) | 0.1166 (2) | 0.4060 (3) | 0.0374 (5) |
| O25 | 0.8522 (3) | 0.0861 (3) | 0.1500 (3) | 0.0846 (8) |
| H25A | 0.856553 | 0.101485 | 0.229323 | 0.127* |
| H25B | 0.946290 | 0.102207 | 0.134009 | 0.127* |
| O26 | 0.0441 (3) | 0.1625 (3) | −0.0146 (3) | 0.0802 (8) |
| H26A | 0.111085 | 0.221718 | 0.026505 | 0.120* |
| H26B | 0.093067 | 0.095594 | −0.054527 | 0.120* |
| C17 | 0.5416 (4) | 0.3186 (3) | 0.7147 (3) | 0.0468 (6) |
| H17 | 0.617209 | 0.385738 | 0.745258 | 0.056* |
| C14 | 0.3246 (3) | 0.1207 (3) | 0.6263 (3) | 0.0494 (7) |
| H14 | 0.251129 | 0.052193 | 0.594172 | 0.059* |
| C15 | 0.3544 (4) | 0.1739 (3) | 0.7538 (3) | 0.0562 (8) |
| H15 | 0.300440 | 0.142249 | 0.808812 | 0.067* |
| C24 | 0.2776 (3) | 0.0157 (3) | 0.3436 (3) | 0.0501 (7) |
| H24 | 0.213652 | −0.022467 | 0.388091 | 0.060* |
| C21 | 0.4690 (4) | 0.1287 (3) | 0.2175 (3) | 0.0471 (6) |

**Table S2**. Bond lengths for **Compound 1**.

| Atom-Atom | Length/Å | Atom- Atom | Length/Å |
|---|---|---|---|
| Cu1-Cl2 | 2.7562(8) | N11-C7 | 1.300(3) |
| Cu1-N4 | 1.946(2) | N12-C5 | 1.336(3) |
| Cu1-N18 | 2.0309(19) | N20-C19 | 1.361(3) |
| Cu1-N11 | 1.955(2) | N20-C21 | 1.331(3) |
| Cu1-N20 | 2.044(2) | C13-C19 | 1.489(4 |
| N8-C7 | 1.341(3) | C13-C14 | 1.378(4) |
| N8-C9 | 1.460(4) | C19-C24 | 1.387(4) |
| N8-C10 | 1.464(4) | C17-C16 | 1.382(4 |
| N6-C5 | 1.385(3) | C14-C15 | 1.370(4) |
| N6-C7 | 1.386(3) | C15-C16 | 1.381(5) |
| N4-C5 | 1.288(3) | C24-C23 | 1.374(4) |
| N18-C13 | 1.351(3) | C21-C22 | 1.388(4) |
| N18-C17 | 1.347(3) | C22-C23 | 1.362(5) |

**Table S3**. Bond Angles for **Compound 1**.

| Atom-Atom-Atom | Angle/° | Atom-Atom-Atom | Angle/° |
|---|---|---|---|
| N4-Cu1-Cl2 | 97.51(8) | C21-N20-C19 | 118.1(2) |
| N4-Cu1-N18 | 94.52(8) | N4-C5-N6 | 121.8(2) |
| N4-Cu1-N11 | 87.06(9) | N4-C5-N12 | 123.6(2) |
| N4-Cu1-N20 | 167.29(9) | N12-C5-N6 | 114.6(2) |
| N18-Cu1-Cl2 | 91.57(6) | N8-C7-N6 | 116.3(2) |
| N18-Cu1-N20 | 80.11(8) | N11-C7-N8 | 123.9(2) |
| N11-Cu1-Cl2 | 96.29(7) | N11-C7-N6 | 119.8(2) |
| N11-Cu1-N18 | 171.72(9) | N18-C13-C19 | 114.2(2) |
| N11-Cu1-N20 | 96.67(8) | N18-C13-C14 | 121.9(2) |
| N20-Cu1-Cl2 | 94.16(6) | C14-C13-C19 | 123.9(2) |
| C7-N8-C9 | 124.5(2) | N20-C19-C13 | 115.2(2) |
| C7-N8-C10 | 119.4(2) | N20-C19-C24 | 121.9(2) |
| C9-N8-C10 | 115.7(2) | C24-C19-C13 | 122.8(2) |
| C7-N6-C5 | 126.71(19) | N18-C17-C16 | 123.1(3) |
| C5-N4-Cu1 | 127.86(17) | C15-C14-C13 | 119.6(3) |
| C13-N18-Cu1 | 115.76(16) | C14-C15-C16 | 119.6(3) |
| C17-N18-Cu1 | 126.44(17) | C23-C24-C19 | 118.1(3) |
| C17-N18-C13 | 117.8(2) | N20-C21-C22 | 123.0(3) |
| C7-N11-Cu1 | 129.10(17) | C23-C22-C21 | 118.0(3) |
| C19-N20-Cu1 | 114.29(16) | C22-C23-C24 | 120.9(3) |
| C21-N20-Cu1 | 127.50(19) | C15-C16-C17 | 118.0(3) |

**Table S4**. Fractional atomic coordinates and isotropic or equivalent isotropic displacement parameters (Å²) for **Compound 2.**

| | X | y | z | Uiso*/Ueq |
|---|---|---|---|---|
| Cu1 | 0.71286 (4) | 0.68262 (10) | 0.40085 (2) | 0.0266 (2) |
| V2 | 0.95148 (4) | 0.21053 (12) | 0.77701 (3) | 0.0258 (2) |
| O3 | 0.5780 (2) | 0.2371 (6) | 0.24431 (14) | 0.0338 (6) |
| N18 | 0.8797 (2) | 1.0618 (8) | 0.56768 (16) | 0.0364 (8) |
| H18 | 0.895209 | 1.070954 | 0.611129 | 0.044* |
| O19 | 0.5638 (2) | 0.9166 (5) | 0.37357 (13) | 0.0319 (5) |
| H19A | 0.508442 | 0.857080 | 0.333968 | 0.048* |
| H19B | 0.571980 | 1.095987 | 0.363003 | 0.048* |
| O20 | 0.9583 (2) | −0.0180 (6) | 0.71170 (13) | 0.0336 (6) |
| O7 | 0.6426 (2) | 0.3841 (6) | 0.34934 (13) | 0.0328 (6) |
| O21 | 0.8321 (3) | 0.3158 (8) | 0.7599 (2) | 0.0621 (11) |
| N8 | 0.6923 (2) | 0.5624 (7) | 0.48928 (15) | 0.0301 (6) |
| N6 | 0.7568 (3) | 0.7441 (7) | 0.31774 (16) | 0.0309 (6) |
| H6A | 0.761922 | 0.911580 | 0.310962 | 0.037* |
| H6B | 0.818598 | 0.671499 | 0.322041 | 0.037* |
| C9 | 0.7508 (3) | 0.6896 (8) | 0.54480 (19) | 0.0312 (8) |
| N15 | 0.8077 (2) | 0.9312 (6) | 0.46295 (15) | 0.0285 (6) |
| C5 | 0.6752 (3) | 0.6282 (7) | 0.26056 (17) | 0.0292 (7) |
| H5A | 0.705772 | 0.576026 | 0.225537 | 0.035* |
| H5B | 0.621080 | 0.753855 | 0.241225 | 0.035* |
| C4 | 0.6277 (3) | 0.3978 (7) | 0.28474 (17) | 0.0264 (6) |
| C16 | 0.8714 (3) | 1.1377 (9) | 0.4610 (2) | 0.0339 (7) |
| H16 | 0.882079 | 1.209382 | 0.422362 | 0.041* |
| O22 | 0.9773 (4) | 0.0592 (7) | 0.84923 (16) | 0.0569 (9) |
| C17 | 0.9163 (3) | 1.2180 (8) | 0.5270 (2) | 0.0365 (8) |
| H17 | 0.962892 | 1.353643 | 0.541011 | 0.044* |
| C12 | 0.6186 (4) | 0.3148 (10) | 0.5606 (2) | 0.0444 (10) |
| H12 | 0.572563 | 0.186789 | 0.564790 | 0.053* |
| C14 | 0.8147 (3) | 0.8906 (8) | 0.52742 (17) | 0.0294 (7) |
| C13 | 0.6282 (3) | 0.3780 (9) | 0.4978 (2) | 0.0377 (8) |
| H13 | 0.588376 | 0.288458 | 0.459888 | 0.045* |
| C10 | 0.7464 (3) | 0.6355 (10) | 0.6094 (2) | 0.0402 (9) |
| H10 | 0.787591 | 0.724344 | 0.646940 | 0.048* |
| C11 | 0.6777 (4) | 0.4430 (10) | 0.6163 (2) | 0.0466 (10) |
| H11 | 0.672426 | 0.402222 | 0.659001 | 0.056* |

**Table S5.** Bond lengths for **Compound 2.**

| Atom-Atom | Length/Å | Atom- Atom | Length/Å |
|---|---|---|---|
| Cu1-N2 | 1.9884(17) | N2-C7 | 1.375(3) |
| Cu1-O1 | 1.9609(15) | N3-C6 | 1.346(3) |
| Cu1-N4 | 1.9925(18) | N3-C8 | 1.369(3) |
| Cu1-O3 | 2.2633(17) | C9-C10 | 1.512(3) |
| Cu1-N1 | 2.0253(16) | C9-O1 | 1.292(2) |
| V1-O4 | 1.6276(18) | C9-O2 | 1.234(2) |
| V1-O5 | 1.8325(16) | C10-N4 | 1.478(3) |
| V1-O5$^{1}$ | 1.8224(15) | O5-V1$^{2}$ | 1.8225(15) |
| V1-O6 | 1.6244(19) | C2-C1 | 1.383(3) |
| C5-C6 | 1.457(3) | C2-C3 | 1.367(4) |
| C5-C4 | 1.380(3) | C8-C7 | 1.374(3) |
| C5-N1 | 1.356(3) | C1-N1 | 1.338(3) |
| N2-C6 | 1.333(3) | C3-C4 | 1.379(4) |

**Table S6**. Bond Angles for **Compound 2.**

| Atom-Atom-Atom | Angle/° | Atom-Atom-Atom | Angle/° |
|---|---|---|---|
| N2-Cu1-N4 | 98.91(7) | C7-N2-Cu1 | 140.96(15) |
| N2-Cu1-O3 | 99.04(7) | C6-N3-C8 | 107.59(18) |
| N2-Cu1-N1 | 81.75(7) | O1-C9-C10 | 115.88(17) |
| O1-Cu1-N2 | 167.50(7) | O2-C9-C10 | 120.80(18) |
| O1-Cu1-N4 | 82.75(7) | O2-C9-O1 | 123.31(19) |
| O1-Cu1-O3 | 92.87(7) | N4-C10-C9 | 109.57(16) |
| O1-Cu1-N1 | 94.02(7) | C9-O1-Cu1 | 114.75(13) |
| N4-Cu1-O3 | 99.42(7) | N2-C6-C5 | 119.34(18) |
| N4-Cu1-N1 | 167.74(8) | N2-C6-N3 | 110.6(2) |
| N1-Cu1-O3 | 92.54(6) | N3-C6-C5 | 129.99(18) |
| O4-V1-O5 | 107.50(9) | V1$^{2}$-O5-V1 | 125.87(8) |
| O4-V1-O5$^{1}$ | 108.49(9) | C3-C2-C1 | 119.5(2) |
| O51-V1-O5 | 115.09(3) | C10-N4-Cu1 | 107.10(13) |
| O6-V1-O4 | 107.04(13) | N3-C8-C7 | 106.8(2) |
| O6-V1-O5$^{1}$ | 108.79(9) | C8-C7-N2 | 108.3(2) |
| O6-V1-O5 | 109.63(10) | N1-C1-C2 | 121.7(2) |
| C4-C5-C6 | 125.7(2) | C2-C3-C4 | 119.5(2) |
| N1-C5-C6 | 112.29(17) | C3-C4-C5 | 118.7(2) |
| N1-C5-C4 | 122.0(2) | C5-N1-Cu1 | 114.18(14) |
| C6-N2-Cu1 | 112.35(15) | C1-N1-Cu1 | 127.22(15) |
| C6-N2-C7 | 106.68(18) | C1-N1-C5 | 118.60(18) |

**Table S7**. Compiled Bonds (Å) and Angles (°) for Selected Extended Vanadate Structures.

| | $NaVO_3$ | $NH_4VO_3$ | $KVO_3$ | Compound 2 |
|---|---|---|---|---|
| VO1 | 1.653(1) | 1.640(4) | 1.643(6) | 1.6276(18) |
| VO2 | 1.643(1) | 1.647(4) | 1.639(6) | 1.6244(19) |
| VO3 | 1.882(3) | 1.803(1) | 1.806(2) | 1.8224(15) |
| VO4 | - | - | - | 1.8325(16) |
| O1-V-O2 | 108.45(7) | 107.3(1) | 110.1(3) | 107.50(9) |
| O1-V-O3 | 135.75(6) | 111.0(3) | 111.5(2) | 108.49(9) |
| O2-V-O3 | 115.60(7) | 110.2(2) | 110.0(2) | 115.09(3) |
